# The weakest point of cardiac resynchronization therapy: new technologies facing old terminology

**DOI:** 10.3389/fcvm.2023.1236369

**Published:** 2023-08-10

**Authors:** Lina Marcantoni, Gianni Pastore, Mauro Biffi, Francesco Zanon

**Affiliations:** ^1^ Arrhythmia and Electrophysiology Unit, Cardiology Department, Santa Maria Della Misericordia Hospital, Rovigo, Italy; ^2^ Istituto di Cardiologia, IRCCS Azienda Ospedaliero Universitaria di Bologna, Bologna, Italy

**Keywords:** cardiac resychronisation therapy, conduction system pacing, left bundle branch pacing, left bundle branch block, biventricular pacemaker therapy

## Abstract

Patients with symptomatic heart failure (HF) and left bundle branch block (LBBB) are currently treated with biventricular pacing (BiV) which has a Class IA recommendation. Given the possibility to re-establish the inter and intra-ventricular synchrony, BiV is commonly referred to as cardiac resynchronization therapy (CRT). This wording is widely utilized and over time the terms BiV and CRT have become interchangeable. Conduction system pacing (CSP) is emerging as a valid therapeutic opportunity to obtain CRT restoring the native conduction via the Purkinje network. Therefore the acronym CRT is no longer synonymous with BiV only but could also refer to CSP. A terminology update is needed to include the resource of CSP to ensure better communication among all the stakeholders involved in managing recipients of cardiac devices and should be a fundamental step in advancing the quality of patient care. Making use of the NBG code to describe the implantable cardiac device would ease such terminology update, since only the first three positions of the five letters NBG code are commonly utilized, while the last two are rarely used.

Patients with symptomatic heart failure (HF) and left bundle branch block (LBBB) are currently treated with biventricular pacing (BiV) which has a Class IA recommendation given the morbidity and mortality benefit (1). As LBBB produces electrical delay in the left ventricular (LV) lateral wall, timed pacing from the left and right leads, or device-based fusion optimization of LV stimulation with intrinsic conduction can re-establish the inter and intra-ventricular synchrony (2–4). Moreover, LV-only with fusion has proven to be as effective as BiV or slightly, though not statistically significant, superior in the setting of LBBB with preserved AV conduction (3). Based on these mechanisms, BiV is referred to as cardiac resynchronization therapy (CRT). This wording is widely utilized and over time the terms BiV and CRT have become interchangeable. Indeed, CRT is a more comprehensive term that comprises the whole setting of simultaneous and non-simultaneous BiV stimulation as well as LV-only pacing. Recently conduction system pacing (CSP) has emerged as a new therapeutic opportunity, and gained relevance given the possibility to restore the native conduction via the Purkinje network and resynchronize LV activation in LBBB patients (2). While BiV aims to reduce LV dyssynchrony, CSP aims to restore the normal cardiac electrical activation by stimulation below the site of the conduction block. His bundle pacing (HBP) theoretically enables both RV and LV synchrony, while several studies have indicated that left bundle branch pacing (LBBP) can achieve LV activation times and synchronicity to the same extent as HBP, especially when the AV interval has been tailored (2, 6, 7). Preliminary studies have shown promising results in terms of QRS narrowing, LV ejection fraction (EF) and NYHA class improvement when CSP is utilized in lieu of CRT (2). Dedicated tools recently introduced in the market have increased the procedural success rate and decreased the need for lead revision during follow-up. Experience with CSP has been growing along with the number of related publications. Although there are still limited randomized studies available, several are ongoing. However, CSP lacks its own terminology and BiV or CRT has been used in its place, possibly generating some confusion because now the acronym CRT is no longer synonymous with BiV only but could also refer to CSP. When referring to CRT, physicians often try to clarify the modality of CRT being delivered by terms as: “conventional CRT”, “traditional CRT”, “classic CRT”, “standard CRT”, all meaning the presence of a coronary sinus lead with simultaneous biventricular stimulation, while “tailored CRT” or “LV-only CRT” qualify settings with non-simultaneous VV delays or LV-only CRT. Therefore, “His resynchronization”, “His-CRT”, “LBBP-CRT” or “physiologic CRT” are used to mean a narrow QRS obtained by CSP in patients with baseline LBBB. “HOT-CRT” and “LOT-CRT” are terms utilized when CSP is combined with a CS lead to achieve resynchronization in case of peripheral LBBB or when an intraventricular conduction delay (IVCD) coexists (2). By these modalities it is indeed possible to correct both conduction system and intraventricular conduction delays to achieve nearly normal synchronization between atria and ventricles. Beyond CRT patients, pacemaker recipients with LBBB and normal or mildly reduced systolic function can obtain restored synchrony by CSP (Figure 1), mimicking the CRT effect similarly to the BLOCK-HF trial (8). Furthermore, HF patients with right bundle branch block (RBBB) and systolic dysfunction can be effectively treated by CSP rather than BiV (9). Shouldn’t we also consider this a CRT? CSP is rapidly spreading, and several trials are ongoing, many of which compare CSP and BiV. HF is highly prevalent in the general population and the incidence of three chamber devices implantation continue to increase, both initial insertion and upgrade (10). As not all patients may need BiV or may succeed in achieving optimal resynchronization by BiV, while the hybridization of CSP and BiV is growing (11), the term CRT should not be used interchangeably with BiV. The term CRT reflects restoration of electrical synchronicity between atria and ventricles, and as such needs to be revised in order to be comprehensive of all the potential pacing modalities capable to achieve this ultimate goal in the different types of atrio-ventricular and inter/intraventricular electrical derangements: AV block, bundle branch block of any type, intraventricular conduction delay. We can expect that Cardiology Societies will work on terminology revision soon and update it to include the resource of CSP. A terminology update is likely to have important consequences for daily practice and clinical research. Consistent nomenclature and terminology will ensure better communication among physicians, nurses and all the stakeholders involved in managing recipients of cardiac devices and should be a fundamental step in advancing the quality of patient care. Physicians can accurately interpret pacemaker EKG to properly identify either correct function or malfunction, and thereby take care of patients safely and effectively. Making use of the NBG code to describe the implantable cardiac device would ease such terminology update, since only the first three positions of the five letters NBG code are commonly utilized, while the last two are rarely used (12). It could be speculated that one position in the code could be used to identify the type of pacing lead implanted, such as “S” for standard myocardial pacing, “C” for conduction system pacing, “L” for leadless pacing, and “B” for the presence of a coronary sinus (CS) lead. Another position could indicate the number of channels available in the device, with “1” representing a single chamber device, “2” representing a dual chamber, and “3” representing a triple chamber device. Based on the given information, the case report in Figure 1 Panel A would be described as DDD-C3, while Panel B would be described as DDD-C2.

**Figure 1:**
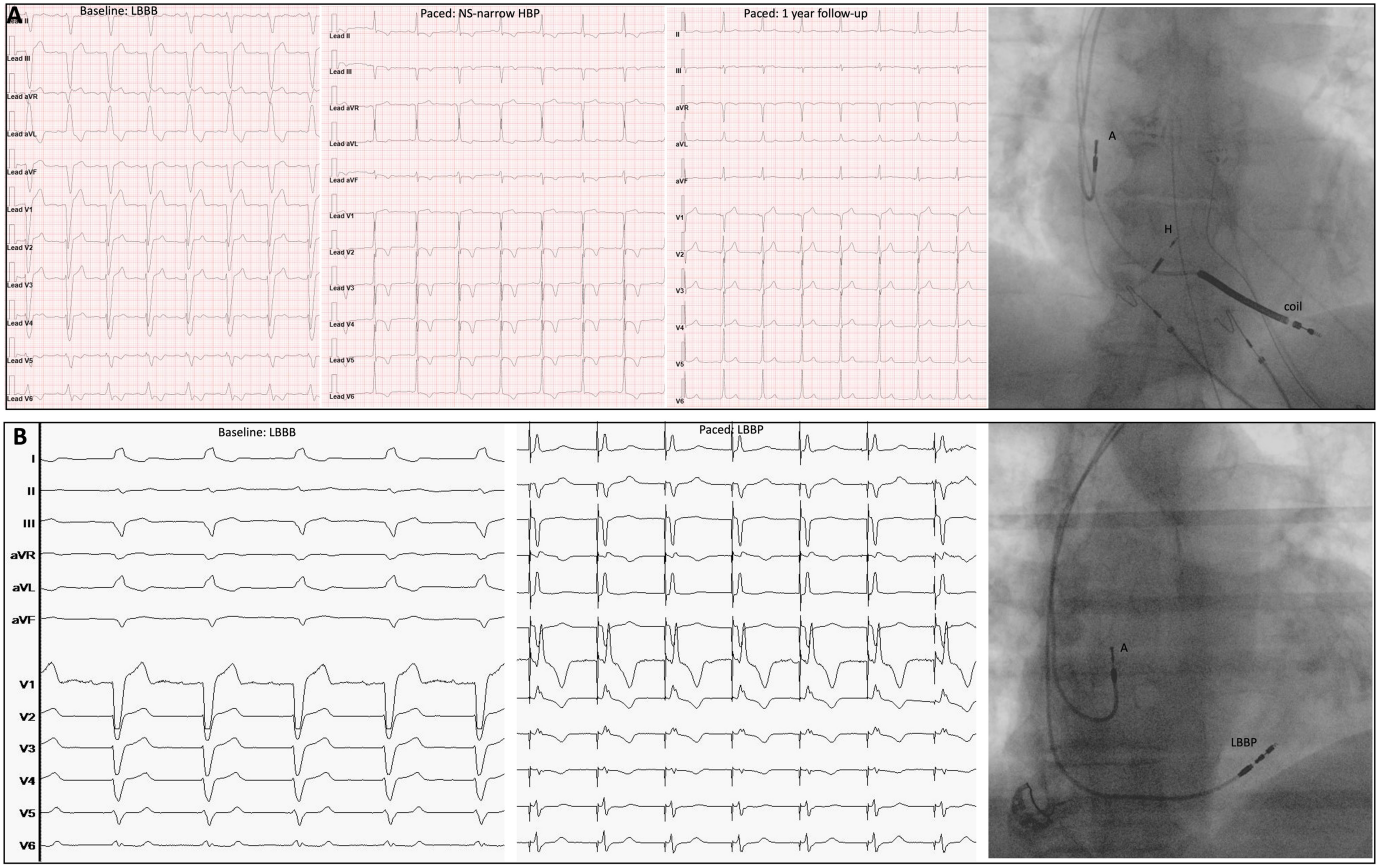
Two different CRTs. Panel (**A**): Male, non-ischemic cardiopathy, EF 32%, LBBB. CRT obtained by non-selective narrow HBP which provided LBBB correction. Baseline EKG, postimplant EKG and 1-year follow-up EKG are showed in sequence at 25 mm/sec. Fluoroscopy shows the final lead position (A: atrial lead; H: His lead; Coil: coil lead). The HBP lead (H) was connected to the LV port on a three-chamber device. Panel (**B**): Male, ischemic cardiopathy, EF 42%, syncope, LBBB. CRT was obtained by LBBP with stylet-driven lead. Baseline EKG and post-implant EKG are showed in sequence, at 25 mm/sec. Fluoroscopy image shows the final lead position. The LBBP lead (LBBP) was connected to the RV port on a dual chamber device.

## Data Availability

The original contributions presented in the study are included in the article/Supplementary Material, further inquiries can be directed to the corresponding author.

## References

[1] McDonagh TA Metra M Adamo M Gardner RS Baumbach A Böhm M ESC Guidelines for the diagnosis and treatment of acute and chronic heart failure: developed by the task force for the diagnosis and treatment of acute and chronic heart failure of the European society of cardiology (ESC). With the special contribution of the heart failure association (HFA) of the ESC. Eur J Heart Fail. (2022) 24(1):4–131. 10.1002/ejhf.2333 35083827

[2] Herweg B Welter-Frost A Vijayaraman P. The evolution of cardiac resynchronization therapy and an introduction to conduction system pacing: a conceptual review. Europace. (2021) 23(4):496–510. 10.1093/europace/euaa264 33247913

[3] Wilkoff B. Adaptive versus conventional cardiac resynchronization therapy in patients with heart failure. Primary results from the AdaptResponse global randomized trial. EHRA Congress 2023. Session: Late Breaking Science-Day2.

[4] Singh JP Cha Y-M Lunati M Chung ES Li S Smeets P Real-world behavior of CRT pacing using the AdaptivCRT algorithm on patient outcomes: effect on mortality and atrial fibrillation incidence. J Cardiovasc Electrophysiol. (2020) 31(4):825–33. 10.1111/jce.14376 32009263 PMC7187461

[5] Strocchi M Lee A WC Neic A Bouyssier J Gillette K Plank G His-bundle and left bundle pacing with optimized atrioventricular delay achieve superior electrical synchrony over endocardial and epicardial pacing in left bundle branch block patients. Heart Rhythm. (2020) 17(11):1922–9. 10.1016/j.hrthm.2020.06.028 32603781

[6] Wang Y Zhu H Hou X Wang Z Zou F Qian Z Randomized trial of left bundle branch vs biventricular pacing for cardiac resynchronization therapy. J Am Coll Cardiol. (2022) 80(13):1205–16. 10.1016/j.jacc.2022.07.019 36137670

[7] Heckman LIB Luermans JGLM Curila K Van Stipdonk AMW Westra S Smisek R Comparing ventricular synchrony in left bundle branch and left ventricular septal pacing in pacemaker patients. J Clin Med. (2021) 10(4):822. 10.3390/jcm10040822 33671420 PMC7923157

[8] Curtis AB Worley SJ Chung ES Li P Christman SA Sutton MSJ. Improvement in clinical outcomes with biventricular versus right ventricular pacing: the BLOCK HF study. J Am Coll Cardiol. (2016) 67(18):2148–57. 10.1016/j.jacc.2016.02.051 27151347

[9] Sharma PS Naperkowski A Bauch TD Chan JIS Arnold AD Whinnett ZI Permanent his bundle pacing for cardiac resynchronization therapy in patients with heart failure and right bundle branch block. Circ Arrhythm Electrophysiol. (2018) 11(9):e006613. 10.1161/CIRCEP.118.006613 30354292

[10] Vaidya VR Asirvatham R Kowlgi GN Dai MY Cochuyt JJ Hodge DO Trends in cardiovascular implantable electronic device insertion between 1988 and 2018 in olmsted county. JACC Clin Electrophysiol. (2022) 8(1):88–100. 10.1016/j.jacep.2021.06.006 34454890 PMC9339254

[11] Jastrzebski M Kielbasa G Cano O Curila K Heckman L De Pooter J Left bundle branch area pacing outcomes: the multicentre European MELOS study. Eur Heart J. (2022) 43(40):4161–73. 10.1093/eurheartj/ehac445 35979843 PMC9584750

[12] Bernstein AD Daubert J-C Fletcher RD Hayes DL Luderitz B Reynolds DW The revised NASPE/BPEG generic code for antibradycardia, adaptive-rate, and multisite pacing. North American society of pacing and electrophysiology/British pacing and electrophysiology group. Pacing Clin Electrophysiol. (2002) 25(2):260–4. 10.1046/j.1460-9592.2002.00260.x 11916002

